# Aspiration of Barium Contrast

**DOI:** 10.1155/2014/215832

**Published:** 2014-09-21

**Authors:** Cristina Fuentes Santos, Bárbara Steen

**Affiliations:** ^1^Unidad de Medicina Interna, Hospital Universitario Fundación Alcorcón, Madrid, Spain; ^2^Unidad de Neumología, Hospital Universitario Fundación Alcorcón, Madrid, Spain

## Abstract

The aspiration of barium contrast is a rare complication that may occur during studies of the digestive tract. Barium is an inert material that can cause anywhere from an asymptomatic mechanical obstruction to serious symptoms of respiratory distress that can result in patient death. We present the case of a 79-year-old male patient in whom we observed the presence of contrast medium residue in the lung parenchyma as an incidental finding during hospitalization. When the patient's medical file was reviewed, images were found of a barium swallow study that the patient had undergone months earlier, and we were able to observe the exact moment of the aspiration of the contrast material. The patient had been asymptomatic since the test.

## 1. Introduction

Pulmonary aspiration is a common event that can occur even in healthy asymptomatic individuals. Although it may go unnoticed and cause no further consequences, on certain occasions, inflammatory changes can occur in the lungs, which end up causing aspiration pneumonia. Some predisposing factors include low level of consciousness, swallowing disorders due to neurological deficiencies, or alterations in esophageal motility. Occasionally, aspiration pneumonia can be caused by fluids that are not toxic themselves to the lungs but can cause chemical pneumonitis or mechanical obstruction of the airways. This occurs with liquids like barium contrast medium. Upper gastrointestinal series with barium swallow is a simple, common test used to study the morphology of the digestive tract. Although rare, occasionally, there is aspiration of the contrast material into the airways. There is not much evidence about the proper treatment of these situations. Even though these aspirations do not usually put the lives of patients at risk because barium is an inert material, we have found several case reports in the literature that have resulted in the death of the patients, who are usually seniors.

We present the case of a 79-year-old male with an incidental finding of barium contrast in the pulmonary parenchyma observed on a chest radiograph.

## 2. Case Presentation

The patient is a 79-year-old male with a history of smoking, hypertension, dyslipidemia, chronic atrial fibrillation, and stage 3 chronic kidney disease, with no baseline cognitive impairment. His surgical history included infrarenal aneurysm repair with the placement of an aortic bifemoral bypass 2 years before the current hospitalization; during the procedure, he presented hemodynamic shock with acute renal failure and mesenteric ischemia that required left colectomy; the postoperative evolution was torpid, with perforation of the cecum, which required resection and placement of a terminal ileostomy. Because of this condition, the patient was being followed-up by the general surgery and gastrointestinal surgery departments.

The patient came to the Emergency Department with acute symptoms of intense dyspnea and palpitations, with no chest pain or vagal response. Upon arrival at the hospital, the patient presented BP 177/76 mm Hg, 95 bpm, tachypnea (30 breaths per min) with signs of supraclavicular retractions and baseline oxygen saturation 85%. Jugular venous pressure was elevated. Lung auscultation detected crepitations in both lung bases. Cardiac auscultation detected arrhythmia, with no murmurs. No edema was seen in the lower extremities. Electrocardiogram confirmed that the patient was in atrial fibrillation with no signs of ischemia, and chest radiograph was compatible with acute pulmonary edema. Transthoracic echocardiogram showed a dilated left ventricle with moderate-severe systolic dysfunction with an estimated ejection fraction of 35% (hypokinetic). Lab analyses were anodyne except for troponin I levels of 3.06 ng/mL. The patient was admitted to the cardiology unit with a diagnosis of heart failure and was treated with oxygen, diuretics, and vasodilators, after which he showed clinical, radiological, and functional improvements.

**Figure 1:**
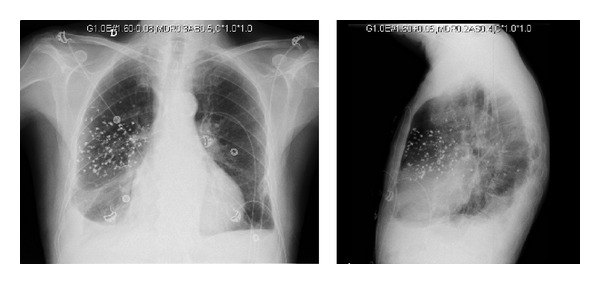
Chest radiograph in posteroanterior and lateral projections.

**Figure 2:**
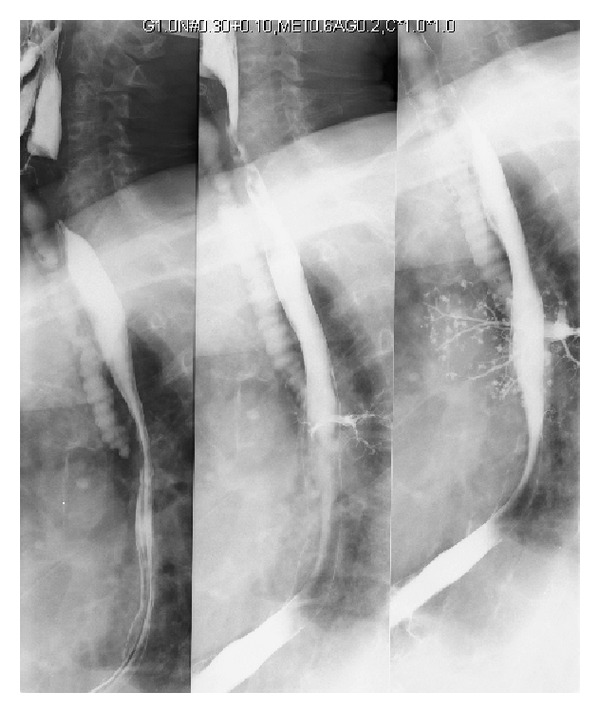
Images of the moment of the barium contrast aspiration during the gastrointestinal series.

During hospitalization, our Pulmonology Department was consulted due to the incidental finding of a multitude of millimetric hyperdense images seen on chest radiograph in the right upper lobe and apical segment of the right lower lobe (Figure 1) related to the remains of barium contrast in the lung parenchyma. We reviewed the clinical history of the patient. Six months before this finding, the patient had a follow-up visit with the general surgery department, at which time he reported presenting a purulent discharge through the ileostomy. An upper gastrointestinal series with barium contrast was done to rule out the existence of an intestinal fistula (Figure 2). During the test, the patient had inhaled barium contrast medium into the bronchial tree. When we interviewed the patient, he denied having had any respiratory symptoms either at that time or during the following months (no cough, expectoration, respiratory noises, or dyspnea) up until the time of his hospitalization in the cardiology unit. The patient continued to be asymptomatic and developed no later complications.

## 3. Discussion

The aspiration of barium contrast is a rare complication of gastrointestinal studies [1]. Barium sulfate is an inert material that does not usually cause chemical pneumonitis and, in cases in which this does occur, it is due to the simultaneous aspiration of gastric content [2]. The severity of the airflow obstruction will depend on the amount of contrast medium that enters the respiratory tract. If the amount is small, there may be no symptomatic effects, as in the case we present. Nevertheless, when there is aspiration of large amounts of contrast material, it interferes with the gas exchange because the barium occupies the alveolar space, leading to a shunt effect and altered ventilation/perfusion (V/Q) ratio with secondary respiratory failure, which can put the patient's life at risk [3]. There have also been reports of cases with severe inflammatory reactions of the bronchial wall after the aspiration of contrast material, which have led to patient death [1, 4]. According to the published cases, there seems to be greater morbidity and mortality in elderly patients with this complication [4–6]. The main symptom that these patients present is cough. Respiratory infection symptoms can appear later on if there has been simultaneous gastric content aspiration.

Inhalation of barium into the airways results in the accumulation of this substance in the bronchoalveolar spaces and the visualization of radio-opacities of varying sizes on chest radiography. The location of the lesions depends on the patient's position at the time of the aspiration [7]. At the structural level, there have been long-term descriptions of thickened interlobular septa, subpleural cysts, and centrilobular micronodules along with barium particles in a subpleural distribution in HRCT [8].

It seems clear that dysphagia and cognitive impairment are predisposing factors for this type of episodes [9], the same as head and neck malignancy and its treatment (surgical and radiotherapy). Since barium swallow tests are done in patients with symptoms of dysphagia, swallowing disorders, and so forth, in whom, at the same time, there is a greater risk of complications, these factors should be recognized before initiating this type of study. Also, the benefits/risks of performing this type of tests should be assessed and preventive measures taken [7].

The prognosis varies depending on the amount of barium contrast that has been inhaled. As for the management of these patients, there is no evidence to determine which treatment is most indicated or appropriate. It seems reasonable to think that, in cases with abundant contrast aspiration that involves impaired respiratory function, bronchoscopy should be done in order to eliminate as much of the contrast as possible from the airway. This entails controlling the evolution of patient oxygen levels. However, bronchoalveolar lavage does not seem to be indicated since it could cause the contrast to spread to other lung areas that were not initially affected [7]. The role of physiotherapy in promoting clearance of barium from the lungs is probably beneficial if aspiration is recognized during or shortly after the study. When secondary respiratory infection is suspected due to clinical, analytical, or radiological parameters, antibiotic therapy should be initiated with coverage for anaerobic microorganisms.

## 4. Conclusion

There is no quality evidence available about the actual incidence of contrast aspiration, treatment, or posterior evolution. Although in some patients few, if any, symptoms may be seen, in others it may cause severe complications and even lead to death. It is essential to try to prevent these episodes since these gastrointestinal series are done in patients with swallowing problems who are more susceptible to bronchial aspiration. In cases with aspirations of large amounts of contrast, bronchoscopy should be done to try to suction as much contrast medium as possible.

In any event, it is important to closely control patient evolution after a contrast aspiration episode, especially in elderly patients as they seem to be more prone to having severe complications.

## References

[1] Kaira K, Takise A, Goto T, Horie T, Mori M (2004). Barium sulphate aspiration. *The Lancet*.

[2] Varatharaj A, Roome C, Allsup S (2012). Barium aspiration. *QJM*.

[3] Chiu C-Y, Wong K-S, Tsai M-H (2005). Massive aspiration of barium sulfate during an upper gastrointestinal examination in a child with dysphagia. *International Journal of Pediatric Otorhinolaryngology*.

[4] Fruchter O, Dragu R (2003). A deadly examination. *The New England Journal of Medicine*.

[5] Gray C, Sivaloganathan S, Simpkins KC (1989). Aspiration of high-density barium contrast medium causing acute pulmonary inflammation: report of two fatal cases in elderly women with disordered swallowing. *Clinical Radiology*.

[6] Gernez Y, Barlési F, Doddoli C (2005). Acute respiratory distress syndrome following inhalation of barium sulfate. *Revue des Maladies Respiratoires*.

[7] Tamm I, Kortsik C (1999). Severe barium sulfate aspiration into the lung: clinical presentation, prognosis and therapy. *Respiration*.

[8] Voloudaki A, Ergazakis N, Gourtsoyiannis N (2003). Late changes in barium sulfate aspiration: HRCT features. *European Radiology*.

[9] Martín MM, Martínez PG (2010). Barium aspiration pneumonitis in an elderly patient under investigation for progressive dysphagia. *Revista Espanola de Geriatria y Gerontologia*.

